# Interfacial Electrostatic Self‐Assembly of Amyloid Fibrils into Multifunctional Protein Films

**DOI:** 10.1002/advs.202206867

**Published:** 2023-01-25

**Authors:** Yangyang Han, Yiping Cao, Jiangtao Zhou, Yang Yao, Xiaodong Wu, Sreenath Bolisetty, Michael Diener, Stephan Handschin, Canhui Lu, Raffaele Mezzenga

**Affiliations:** ^1^ State Key Laboratory of Polymer Materials Engineering Polymer Research Institute of Sichuan University Sichuan 610065 P. R. China; ^2^ ETH Zurich Department of Health Science and Technology Schmelzbergstrasse 9, LFO E23 Zurich 8092 Switzerland; ^3^ BluAct Technologies GmbH Zurich 8092 Switzerland

**Keywords:** amyloid fibrils, amyloid film, electrostatic interaction, magnetic sensor, self‐assembly

## Abstract

Amyloid fibrils have generated steadily increasing traction in the development of natural and artificial materials. However, it remains a challenge to construct bulk amyloid films directly from amyloid fibrils due to their intrinsic brittleness. Here, a facile and general methodology to fabricate macroscopic and tunable amyloid films via fast electrostatic self‐assembly of amyloid fibrils at the air–water interface is introduced. Benefiting from the excellent templating properties of amyloid fibrils for nanoparticles (such as conductive carbon nanotubes or magnetic Fe_3_O_4_ nanoparticles), multifunctional amyloid films with tunable properties are constructed. As proof‐of‐concept demonstrations, a magnetically oriented soft robotic swimmer with well‐confined movement trajectory is prepared. In addition, a smart magnetic sensor with high sensitivity to external magnetic fields is fabricated via the combination of the conductive and magnetic amyloid films. This strategy provides a convenient, efficient, and controllable approach for the preparation of amyloid‐based multifunctional films and related smart devices.

## Graphical abstract

Multifunctional amyloid films via fast electrostatic self‐assembly of amyloid fibrils at the air–water interface are designed. A magnetically oriented soft robotic swimmer with well‐confined movement trajectory, and smart magnetic sensing devices with high sensitivity are fabricated, which can be used for real‐time detection and continuous monitoring of external magnetic fields.

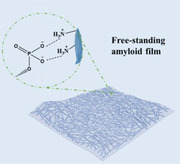

## 1 Introduction

Protein thin films with biocompatibility and biodegradability have garnered considerable interest in materials science, especially for medical and electronic applications.^[^ ^1^, ^2^ ^]^ Self‐assembly can be used as a powerful approach to direct diverse multiscale structures, including protein thin film.^[^ ^3^, ^4^, ^5^ ^]^ Increasing efforts are converging toward improvements of the protein thin films construction, e.g., via cross‐linker‐induced assembly,^[^ ^6^ ^]^ evaporation‐induced assembly,^[^ ^7^ ^]^ or destabilization‐based assembly.^[^ ^8^ ^]^ Interfacial assembly of proteins at the air–water interface mediated by covalent/noncovalent interactions is a facile strategy to form a 2D protein films, due to the specific orientation and concentration enrichment of protein at the interface.^[^ ^9^, ^10^, ^11^ ^]^ Amyloid fibrils, with highly ordered fibrillar morphology and rich surface chemistry, are considered as versatile building blocks for functional materials.^[^ ^12^, ^13^, ^14^, ^15^ ^]^ Amyloid can be generated from a wide range of soluble peptides and proteins, by unfolding and hydrolyzing monomers spontaneously to self‐associate into small oligomers, supramolecular aggregates, and form fibrillar structures. Amyloid with fibrillar structures can be assembled into multiscale materials, such as composite membrane for water purification and three dimensional conductive aerogel with sensing properties.^[^ ^14^, ^16^, ^17^ ^]^ Recently developed amyloid‐like protein films assembled from lysozyme monomers at an interface hold great potentials for water treatment and medical applications.^[^ ^18^, ^19^ ^]^ However, the direct interfacial assembly of amyloid fibrils, instead of protein oligomers, to generate functional amyloid film, is still in its infancy.

Electrostatic assembly of oppositely charged biomacromolecules, such as proteins, polysaccharides, and low molecular weight biomolecules in solution—also referred to as coacervation—is associated, in vivo, with important biological processes such as the formation of intracellular membraneless organelles,^[^ ^20^ ^]^ but can also be engineered in vitro to design innovative materials with potential applications in food,^[^ ^21^ ^]^ pharmaceuticals,^[^ ^22^ ^]^ personal care, and biological scaffolds.^[^ ^23^, ^24^ ^]^ Compared to other biopolymers, such as polysaccharides, use of amyloids for surface electrostatic assembly provides several advantages, i) amyloids cover contours lengths of the order of 10^1^ µm, while maintaining the solution viscosity extremely low; ii) polysaccharides do not possess an isoelectric point (pI), which forces coacervation with only cationic species (or in the unique case of chitosan, anionic species): amyloids allow both cationic and anionic complexation depending on whether the coacervation is carried out above or below the p; iii) chemical and supramolecular manipulation of amyloids is far superior to polysaccharides due to the availability of 20 essential amino acid residues on their surface compared to polysaccharides; and iv) amyloids are well known for the surface activity,^[^ ^25^, ^26^, ^27^ ^]^ compared to polysaccharides.^[^ ^28^ ^]^

In this work, coacervation is utilized for the interfacial assembly of amyloid fibrils by using a low‐molecular‐weight, yet highly charged, coacervating moiety, phytic acid, as an electrostatic gelator. Bypassing the problems associated with high molecular weight gelators, high‐order molecular self‐assemblies can be produced using amyloid‐phytic acid for designing advanced functional protein thin films with tunable physical and functional properties. We demonstrate two possible uses of these electrostatically assembled films as soft magnetic swimmers and magnetic sensors by combining Fe_3_O_4_ magnetic nanoparticles in one case, and the same magnetic nanoparticles in presence of conductive carbon nanotubes (CNTs) in the other case. In both examples, advanced applications can be targeted for the ensued amyloid‐based films, specifically, smart magnetic sensing devices with high sensitivity, which can be used for real‐time detection and continuous monitoring of external magnetic fields.

## 2 Results and Discussion

**Figure 1:**
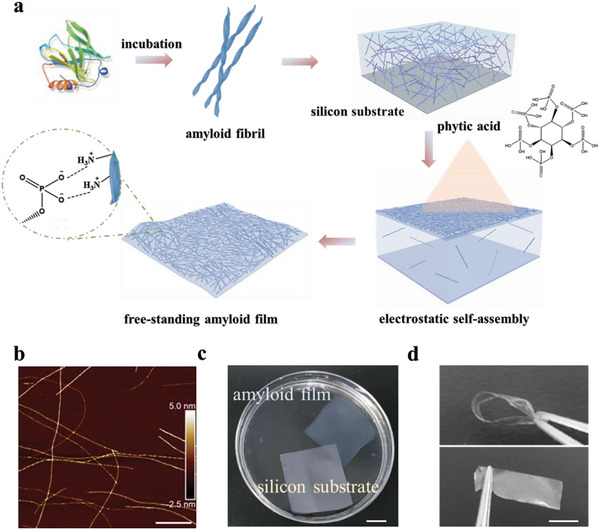
Illustration and fabrication of amyloid film. a) Schematic illustration of the preparation of amyloid film via fast electrostatic self‐assembly of amyloid fibrils at air–water interface. b) AFM height image of BLG fibrils (scale bar: 600 nm). c) Image of the amyloid film (0.2 wt%) floating on the water separated from the original silicone substrate (scale bar: 10 mm). d) Photographs show that the dried BLG film (2 wt%) can be bended and recovered to its pristine shape (scale bar: 10 mm).

Amyloid fibrils possess a propensity toward self‐assembly and form a network at the air‐water interface due to their amphiphilic character.^[^ ^27^, ^29^ ^]^ Our hypothesis for the development of amyloid film was to start by directly assembling the amyloid fibrils at the air‐water interface, as illustrated in **Figure** **1**a. *β*‐lactoglobulin (BLG) was used as a model protein to form semiflexible amyloid fibrils. BLG fibrils were produced from the assembly of BLG monomers, following a previous protocol.^[^ ^12^, ^30^, ^31^ ^]^ The atomic force microscopy (AFM) height image (Figure 1b) shows a high aspect ratio morphology of BLG fibrils, with several nanometers in height and a few micrometers in contour length (i.e., aspect ratio ≈10^3^). Phytic acid, myo‐inositol with the presence of 12 ionizable protons, that can form stable complex coacervation with proteins,^[^ ^32^ ^]^ was introduced as a physical cross‐linker for BLG fibrils via the aerosol‐spraying approach. The diffusion and migration of phytic acid molecules and amyloid fibrils toward the interfacial surface led to the formation of an amyloid thin film at the air–water interface. The amyloid film was easily unleashed as a freestanding film (Figure 1c and Video S1, Supporting Information). After drying at room temperature, the resulting amyloid film is transparent and flexible (Figure 1d). Compared to other molecules shown in Figure S1 (Supporting Information), phytic acid shows the excellent ability to generate intact protein films with amyloid fibrils. It is important to note that, other protein forms such as monomers, or shortened fibrils, are not able to form an intact film at the air–water interface via this approach (Figure S2, Supporting Information). This suggests that the high aspect ratio of protein structures is essential for the formation of freestanding amyloid films. The air–water interface is also a key for directing the amyloid fibrils to self‐assemble into a freestanding thin film as the direct mixing of phytic acid and protein fibrils only causes the formation of large aggregates. In addition to BLG fibrils, this protocol is also applicable to lysozyme fibrils (Figure S3, Supporting Information), indicating the universality of this approach for preparing amyloid films at the air–water interface.

**Figure 2:**
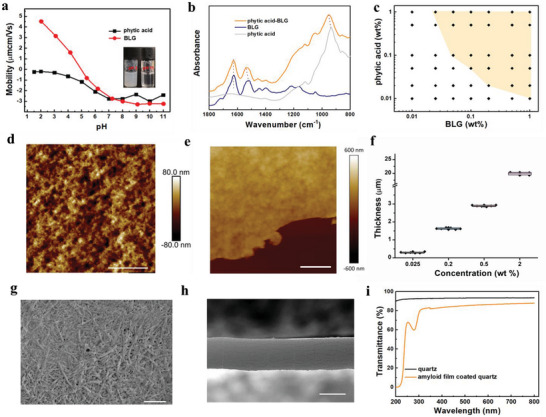
Structure and characterization of amyloid films. a) Electrophoretic mobility of BLG fibrils and phytic acid as a function of pH. Inset photographs showing the mixture of BLG fibrils and phytic acid at pH = 2 and 11. b) FTIR spectra of phytic acid, BLG, and phytic acid‐BLG. c) Plot of the concentrations of phytic acid and BLG fibrils for film formation. The stable film region is in the yellow highlighted area. d,e) AFM images of the surface morphology and the boundary of amyloid film (0.025 wt%) (scale bar: 3 µm). f) The thickness of amyloid films prepared from BLG fibril solution at different concentrations. g,h) SEM image of the surface morphology of an amyloid film (0.025 wt%) (g, scale bar: 1 µm) and cross section of amyloid film (2 wt%) (h, scale bar: 10 µm). i) UV–vis spectra of quartz after coating with amyloid film (1.6 µm thickness).

Zeta potential of phytic acid and BLG fibrils was characterized over a wide pH range (**Figure** **2**a). In the studied pH range, phytic acid is negatively charged. The isoelectric point of BLG fibrils is around pH 5. The overall charge on the surface of protein fibrils is different when the pH of the solution is above or below the isoelectric point. At low pH conditions, BLG fibrils are positively charged, interacting electrostatically with negatively charged phytic acid, resulting in phase separation and formation of aggregates (Figure 2a, inset). At high pH environments, negatively charged BLG fibrils remain stable in suspension while encountering with negatively charged phytic acid, resulting in no film formation at the air‐water surface via the presented approach. This indicates that electrostatic interaction is the dominant driving force for amyloid film formation. Indeed, the resultant amyloid films are stable in different solvents, except in an alkaline solution (Figure S4, Supporting Information). The formed film can be intact in deionized water for 7 d (Figure S5, Supporting Information).

The effect of phytic acid on the BLG fibril structure was studied by Fourier‐transform infrared spectroscopy (FTIR) (Figure 2b). The peak of BLG fibrils at 1700–1600 cm^−1^, reflects the stretching vibration of the moieties C=O in the amide I band, and can provide the information of protein secondary structure.^[^ ^33^ ^]^ After compounding with phytic acid, the characteristic peak in the amide I band does not change significantly (Figures S6 and S7, Supporting Information), indicating that phytic acid has negligible on the conformation of BLG fibrils. The characteristic peak of BLG fibrils in the amide II band region (1500–1600 cm^−1^) attributed to the bending vibration of the N—H bond and the stretching vibration of the C—N bond shifts to a high wavenumber (Figure S6, Supporting Information).^[^ ^34^, ^35^ ^]^ The characteristic peak of phytic acid assigned to the P—O vibration groups also shifts from 935 to 950 cm^−1^.^[^ ^32^, ^36^, ^37^ ^]^ These results indicate that the phosphoric acid groups of phytic acid and the amino groups of the protein generate electrostatic interactions and bind to each other (also stable at high temperatures, Figure S8, Supporting Information), but the highly ordered *β*‐sheet secondary structure of BLG fibrils is preserved.

The assembly behavior of BLG fibrils and phytic acid at the air–water interface under different concentrations was further studied. As shown in Figure 2c, the formation of an amyloid film mainly depends on the concentration of BLG fibrils in the initial solution. When BLG fibrils concentration is lower than 0.025 wt%, the amyloid film will not be formed even if the concentration of phytic acid is increased to 1 wt%. This is because BLG fibrils at low concentration are not able to form a percolating network and generate an intact film. Similarly, when the concentration of phytic acid is lower than a critical value, BLG fibrils cannot fully form a stable film structure. Only when both concentrations of phytic acid and *β*‐lactoglobulin fibrils are above a certain critical value, a free‐standing amyloid film can form at the air–water interface.

The surface morphology of the amyloid film is shown in Figure 2d. The assembly of amyloid fibrils at the interface leads to the formation of a dense network, which is also clearly visible in SEM (Figure 2g). The thickness of thin amyloid films was measured by AFM (Figure 2e). The thickness changes with the concentrations of BLG fibril solution (Figure 2f). At 0.025 wt%, the average thickness of the film is around 0.3 µm. As the concentration of BLG fibrils used became higher, the thickness of the formed film increased correspondingly. At 2 wt% BLG fibrils, the thickness of the film reaches ≈20 µm. The cross section of this thicker film was observed to be uniform, by SEM imaging (Figure 2h). These results indicate that the thickness of amyloid films can be controlled by varying the concentration of the BLG original solution. The concentration of phytic acid can also affect the thickness of the formed protein films as shown in Figure S9 (Supporting Information). In the visible light range (400–700 nm), the amyloid film with 1.6 µm thickness has excellent optical transparency with the percentage transmittance of above 80%, as measured by UV–vis absorption spectroscopy (Figure 2i). Absorption peaks at 280 nm, mainly reflect the *π*–π* transition process of aromatic amino acid residues.^[^ ^38^ ^]^ This thin amyloid film can stably adhere to the surface of various substrates (polyethylene terephthalate [PET], polydimethylsiloxane [PDMS], and polytetrafluoroethylene [PTFE]), to achieve functional modification of the respective substrate surface, for example, the hydrophilicity of these substrates is significantly improved by this coating (Figure S10, Supporting Information).

**Figure 3:**
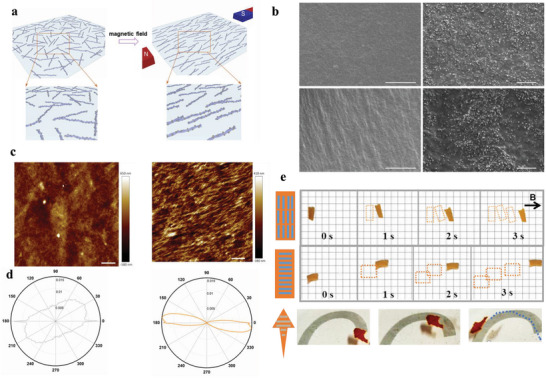
Amyloid film‐based robotic swimmer. a) Schematic of Fe_3_O_4_‐coated BLG fibrils aligned under external magnetic field. b) SEM images of Fe_3_O_4_/BLG films prepared without (top) and with (bottom) external magnetic field with different magnifications (scale bars: 10 µm for left, and 500 nm for right). c,d) AFM images and corresponding orientation distributions of Fe_3_O_4_/BLG films prepared without (left) and with (right) external magnetic field (scale bars: 20 µm). e) Images showing the movement of magnetic film swimmers with different aligned structures under a magnetic field.

This interface assembly strategy has the advantages of controllable preparation and easy removal from the substrates, and can be exploited to construct amyloid films with diverse functionalities. The integration of nanoparticles into thin amyloid films offers numerous advantages to tailoring the structural and functional properties by well‐defined design.^[^ ^30^, ^39^ ^]^ To demonstrate the customized functionalization of this thin film system, amyloid films with integrated Fe_3_O_4_ magnetic nanoparticles were fabricated following the same protocol, by dispersing Fe_3_O_4_ magnetic nanoparticles in charged BLG fibril solutions. These nanoparticles‐modified BLG fibrils show magnetic‐responsive behavior (Figure S11, Supporting Information). By applying external magnetic field, the hybrid nanofibrils are oriented along the direction of the magnetic field. After applying phytic acid, hybrid amyloid films with well‐aligned structures are generated, as illustrated in **Figure** **3**a. SEM images (Figure 3b) clearly show that the application of the external magnetic field guided the aligning of hybrid fibrils in the magnetic field direction. Figure 3c shows the AFM images of hybrid amyloid films with and without exposure to an external magnetic field during the film formation. The alignment of amyloid fibrils decorated with nanoparticles was visible in films prepared in the presence of the magnetic field. Orientation J software was used to analyze the alignment of structures in AFM images (Figure 3d). The orientation distribution of the fibrils in the aligned amyloid film exhibits a narrow‐elongated distribution with a clear peak along one direction (Figure S12, Supporting Information), while amyloid films without an external magnetic field exhibit an isotropic distribution.

Magnetic soft swimmers based on this hybrid amyloid film with directionally aligned inner structures are developed, which can be easily actuated by a magnetic field. Figure 3e shows the responsive behavior of swimmers with different oriented structures in magnetic fields. The movement of the swimmer in water was driven by using an external permanent magnet. The aligned film in the swimmer reoriented itself in response to the applied magnetic field and the swimmers with different aligned structures were driven at an increasing speed in the according direction. When not directly oriented towards the magnet, the film rotated first, then moved towards the magnet (Video S2, Supporting Information). By changing the direction of the external magnetic field, it is possible to guide the swimmer on a desired trajectory of motion. As shown in Figure 3e and Video S3 (Supporting Information), a fish‐shaped swimmer with an aligned fibrillar structure moves along a given trajectory by manually driving it when changing the directions of the magnet manually. Our approach provides an efficient strategy to design magnetic soft swimmers based on hybrid amyloid film with biocompatibility and biodegradability, which may have potential medical applications.

In addition to inorganic nanoparticles, functional films can also be fabricated via the same approach by introducing organic nanomaterials into amyloid fibril solutions. Here, conductive protein‐based films were developed by adding CNTs into the amyloid solution. The conductivities of the CNTs‐amyloid film can be adjusted by changing the content of CNTs (Figure S13, Supporting Information). The CNTs are uniformly distributed in the films without aggregation (Figure S14, Supporting Information). The mechanical performance of the protein films with and without additives was studied by tensile test as shown in Figure S15 (Supporting Information).

**Figure 4:**
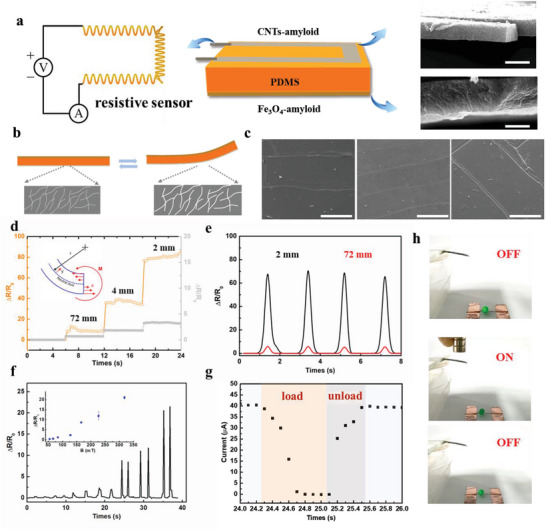
Construction and characterization of magnetic sensors. a) Sandwich structure of the magnetic sensor. SEM images show the cross sections of the conductive layer (top) and magnetic layer (bottom). Scale bars are 5 µm. b,c) Schematics and SEM images of the cracks in the conductive layer under increased bending conditions. The scale bar is 200 µm. d) Resistance changes of the sensor under bending with the radius of 2, 4, and 72 mm (orange color: conductive film as the outer side of bending, gray color: conductive film as the inner side of bending). e) Resistance changes of the sensor under repeated bending with curvature radius of 2 and 72 mm. f) Resistance changes of the sensor under different magnetic field intensities. g) Real‐time response to the load/unload magnetic field. The conductivities recover to the original state within 0.4 s after the removal of the magnetic field. h) Images showing the response of the sensor when connected to a circuit with an LED light.

Combining these findings into a flexible structure of sensors give rise to promising applications in wearable smart systems, such as touchless human–machine interaction and soft robotics.^[^ ^40^, ^41^, ^42^, ^43^ ^]^ The further integration of the above presented magnetic and conductive films, which serve as magneto sensitive and conductive signal transduction materials, respectively, give the basis for the development of flexible sensors for strain and magnetic fields. The structure and fabrication process of such sensors are revealed in **Figure** **4**a and Figure S16 (Supporting Information). The Fe_3_O_4_‐amyloid magnetic film (6.5 µm thickness) as the active layer was first transferred onto the top surface of a flexible PDMS substrate (600 µm). Then the CNTs‐amyloid conductive film (3.3 µm thickness) was transferred onto the other side of the PDMS film, resulting in a sandwich‐structured material.

An electrical resistance sensor could be realized in an effective way by formation of reversible microcracks in the composite conductive film layer (Figure 4b). Parallel microcracks are generated and controlled in conductive thin films by pre‐stretching PDMS from both ends with a controlled strain.^[^ ^44^, ^45^, ^46^, ^47^ ^]^ These microcracks offered the possibility for the reversible change in electrical conductivity of the sensor during bending and releasing cycles.^[^ ^48^, ^49^, ^50^ ^]^ As shown in Figure 4c, SEM images reveal that microcracks in conductive film became wider with an increase in the bending. As a result, the electrical resistance of the sensor was significantly raised during bending with different bending radius of the conductive layer as outer side (Figure 4d). When the deformation was released, the substrate relaxed and caused the shrinking of the microcrack in width, microcrack edges were reconnected, resulting in a decrease of the resistance (Figure 4e). Based on the repeated opening/closing of microcracks, high sensitivity of strain sensors can be achieved.

Highly sensitive magnetic sensors are realized in the same effective way by formation of reversible microcracks in the composite conductive film layer, whose crack widths are sensitively triggered by the moderate deformation derived by external magnetic fields. A microcracks‐based magnetic‐field sensor (MMFS) can then be developed by generating an electrical signal in response to the magnetic‐induced deformation. When a magnet approaches the magnetic layer on the top of MMFS from a distance, it leads to the bending of MMFS. The bending of MMFS triggers the deformation of the conductive layer and facilitate simultaneous read‐out of electrical signals, thereby enabling magnetic sensing.^[^ ^51^ ^]^ The change in resistance of the MMFS under different magnetic fields is shown in Figure 4f. The sensitivity of magnetic sensor is defined as *S* = (Δ*R*/*R*_0_)/Δ*B*, where Δ*R* denotes the relative change in resistance, *R*_0_ is the initial resistance of the sensor, and Δ*B* is the relative change in the magnetic field. The highest sensitivity is 6% mT^−1^. To further assess the sensing performance of the magnetic sensor, we investigated the current change response, which exhibits a fast response and recovery time (Figure 4g). When no magnetic field is loaded, the current remains unchanged. Once a magnetic field is introduced, the current decreases obviously, and the current recovers to the initial state after removal of the magnetic field with a response time of 0.4 s, comparable to previous magnetic sensors.^[^ ^52^, ^53^ ^]^ A demonstration of current change by switching an LED light through a circuit is shown in Figure 4h and Video S4 (Supporting Information). Enabled by this unique sensing mechanism and magnetic‐induced deformation, the MMFS exhibits a fast response and excellent magnetic‐field sensing performance.

## 3 Conclusion

In summary, we report a convenient, efficient, and controllable methodology for fabricating amyloid films starting directly from amyloid fibrils. Using a phytic acid aerosol spraying process, amyloid thin films with uniform thickness can be successfully constructed at the air–water interface based on electrostatic interactions between amyloid fibrils and phytic acid. The thickness of the amyloid film can be effectively controlled via precise regulation of the preparation conditions. By introducing functional nanoparticles (such as conductive CNTs, Fe_3_O_4_ nanoparticles), amyloid films with different functionalities were further developed. A magnetically oriented soft robotic swimmer with well‐confined movement trajectory was successfully prepared. Combining the above‐mentioned conductive and magnetic amyloid films, smart magnetic sensing devices with high sensitivity are fabricated, which can be used for real‐time detection and continuous monitoring of external magnetic fields.

## 4 Experimental Section

Methods and any associated references are available in the Supporting Information.

## Conflict of Interest

The authors declare no conflict of interest.

## Supporting information

Supporting Information

Supplemental Video 1

Supplemental Video 2

Supplemental Video 3

Supplemental Video 4

## Data Availability

The data that support the findings of this study are available from the corresponding author upon reasonable request.
