# Supplementary material for: Interfacial Electrostatic Self‐Assembly of Amyloid Fibrils into Multifunctional Protein Films
Source: Adv Sci (Weinh). 2023 Jan 25;10(9):2206867. doi: 10.1002/advs.202206867 (PMC10037951; doi:10.1002/advs.202206867)
Supplement: Supplementary file 1 — Supporting Information [file ADVS-10-2206867-s001.pdf]

ADVANCED SCIENCE
Open Access

Supporting Information

for *Adv. Sci.*, DOI 10.1002/advs.202206867

Interfacial Electrostatic Self-Assembly of Amyloid Fibrils into Multifunctional Protein Films

Yangyang Han, Yiping Cao, Jiangtao Zhou, Yang Yao, Xiaodong Wu, Sreenath Bolisetty, Michael Diener, Stephan Handschin, Canhui Lu and Raffaele Mezzenga**

Supporting Information

Interfacial Electrostatic Self-assembly of Amyloid Fibrils into Multifunctional Protein Films

Yangyang Han, Yiping Cao, Jiangtao Zhou, Xiaodong Wu, Sreenath Bolisetty, Yang Yao, Michael Diener, Stephan Handschin, Canhui Lu, and Raffaele Mezzenga**

Materials and Methods

Materials

β-lactoglobulin (BLG) was purified from whey protein isolated based on the previous protocol, provided by Fonterra, New Zealand. Phytic acid solution 50 % (w/w) in H_2O was purchased from Sigma-Aldrich. HCl was from VWR International. Magnetic iron nanoparticles, with an average size of 10 nm were purchased from Ferrotec Corporation. Multiwall carbon nanotubes (CNT, 95.0%), with a mean diameter of 10-20 nm and a length of 10-30 µm, were obtained from Chengdu Organic Chemicals Co., Ltd Chinese Academy of Sciences. Other chemicals were purchased from commercial chemical company.

Preparation of amyloid fibrils.

β-lactoglobulin amyloid fibrils were prepared by incubation of 2 wt% protein monomer solution at pH 2 and 90 °C for 5 h with a magnetic stirring of 300 rpm. During the incubation, the protein monomers unfold, hydrolyze and self-assemble into amyloid fibrils. The prepared fibrils were stored at 4 °C for further use.

Fabrication of amyloid thin film.

The desired amount of BLG fibrils (pH 2) was cast onto a hydrophilic substrate (silicon wafer). Then, 2 mL phytic acid (1 wt%, pH around 1.5) solution was sprayed onto the BLG fibrils suspension by using a commercial airbrush at a distance of ∼15 cm. After 5 minutes, the newly formed film was transferred into a petri dish filled with deionized water. The floated films were further transferred to the surface of different substrates and dried at room temperature.

Fabrication of magnetic amyloid thin film.

The hybrid magnetic nanoparticle-coated amyloid fibrils were prepared by mixing the desired amount of magnetic magnetite (Fe_3O_4) nanoparticles and BLG fibrils, according to the previous protocol.[1] Then, following the procedure developed above, the aligned hybrid amyloid film was formed by applying external magnetic fields, generated by placing a permanent magnet (NdFeB). The aligned film was put in a petri dish filled with water. The movement of the film was controlled by displacing a magnet at different positions.

Fabrication of magnetic sensors.

A sandwich-structured magnetic sensor was prepared. A magnetic layer and conductive layer were respectively deposited on the two sides of PDMS (Dow Corning 184, cured at 60 °C for 3 h) respectively. The magnetic layer was formed following the method mentioned above, without an external magnetic field. The conductive layer was prepared as follows: CNTs (0.1 wt%) were added to distilled water and sonicated for 30 min, with BLG monomers as a suspending agent (0.02 wt%). Subsequently, 10 mL CNTs suspension was mixed with 10 mL BLG fibrils suspension (0.4 wt%). The desired amount of CNTs-amyloid suspension was cast onto a silicon substrate and formed a free-standing film by introducing a phytic acid solution. The formed film was transferred onto one surface of PDMS with the other surface coated with the magnetic film and then dried at room temperature. Two ends of the composites were coated with conductive silver paste to eliminate the contact resistance and obtain a steady signal output. Different magnetic intensities are measured by a magnetometer by changing the distance between the magnet and the surface of sensor.

Characterization

Fourier transform infrared spectroscopy (FTIR) was used to characterize the interaction between fibril and phytic acid. FTIR experiments were carried out by a Varian 640 spectrometer equipped with a Golden Gate diamond ATR stage. Samples were scanned over the range from 4000 to 400 cm^{-1} with a resolution of 4 cm^{-1} at room temperature and averaged over 64 scans. The spectra were also recorded at the desired temperature by precise temperature control.

The light transmittance of amyloid film was studied by ultraviolet-visible (UV-vis) absorption spectroscopy (Cary UV/Vis spectrometer (Agilent)). The amyloid films with different initial concentrations of BLG fibrils were transferred to the surface of the quartz plate, and tested after drying.

Zeta potential of amyloid fibrils (0.1 wt%) and phytic acid (0.1 wt%) at different pH determined by the Zetasizer Nano ZS DLS device (Malvern Instruments). Samples were loaded into the disposable folded capillary cells (DTS1070).

Tapping mode atomic force microscopy (AFM) was carried out on a MultiMode VIII Scanning Force Microscope (Bruker) under ambient conditions using a rectangular cantilever with a vibrating frequency of 150 kHz (RTESPA-150) or a triangular cantilever with a vibrating frequency of 70 kHz (ScanAsyst-air). The amyloid films were transferred to a clean glass surface, then the film was cut with a blade, and the AFM probe was moved to the vicinity of the fracture to measure the height change near the fracture. Images were flattened using the NanoScope Analysis 1.5 software.

Scanning electron microscopy (SEM) on a Merlin FE-SEM (Zeiss) was performed to observe the morphology and cross-section of the films. SE-Images (Everhart-Thornley detector) were recorded at a working distance of 6-7 mm and an acceleration voltage of 2 kV. The samples were sputter-coated with 4 nm of platinum/palladium (CCU-10, Safematic) before imaging.

The electrical conductivity of samples was measured at room temperature with a two-probe method using a resistance meter (UT61, Uni-Trend, China). The current signals of sensors were measured in real-time with a Keithley 2601B source meter.

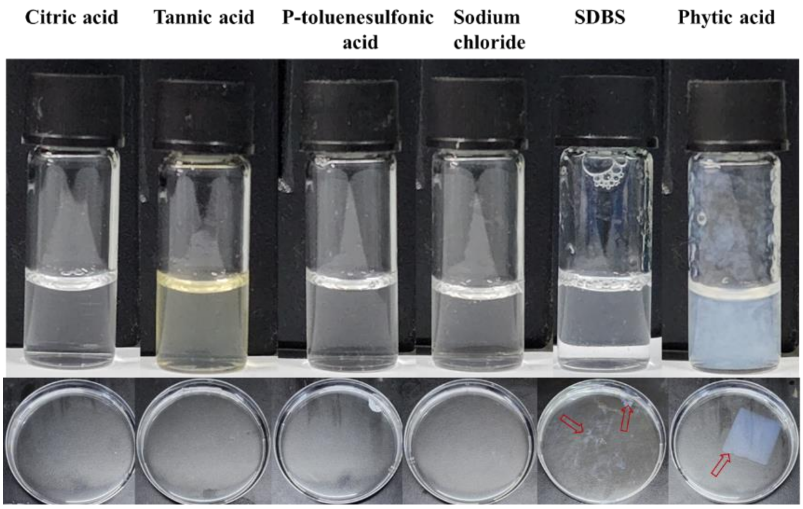

Figure S1. Photographs of solutions (top) of BLG fibrils (0.5 wt%) mixed with different molecules and photographs of films (bottom) that show BLG fibrils to form intact films after spraying these molecules.

Other small molecules were introduced to BLG fibrils (pH 2, 0.5 wt%) by the same approach. Tannic acid (15 mM) and citric acid (15 mM), which are considered as chelating agents with branched group, cannot generate coacervate and form film with BLG fibrils. NaCl (450 mM) as charge screening ion that can be used to make BLG fibril gel, cannot form a stable film at the interface by this method. Also, no film can be observed by P-toluenesulfonic acid (15 mM). SDBS is a typical anionic surfactant. From the images, we can see some aggerates by directly adding SDBS, but no intact films at the interface. We also tried to use anionic polymer, alginate, which generated coacervate with BLG fibrils, however, it is difficult to use a spraying method to introduce this anionic polymer’s viscous solutions, and also the migration rate of polymer in solution is much slower, which is not effective.

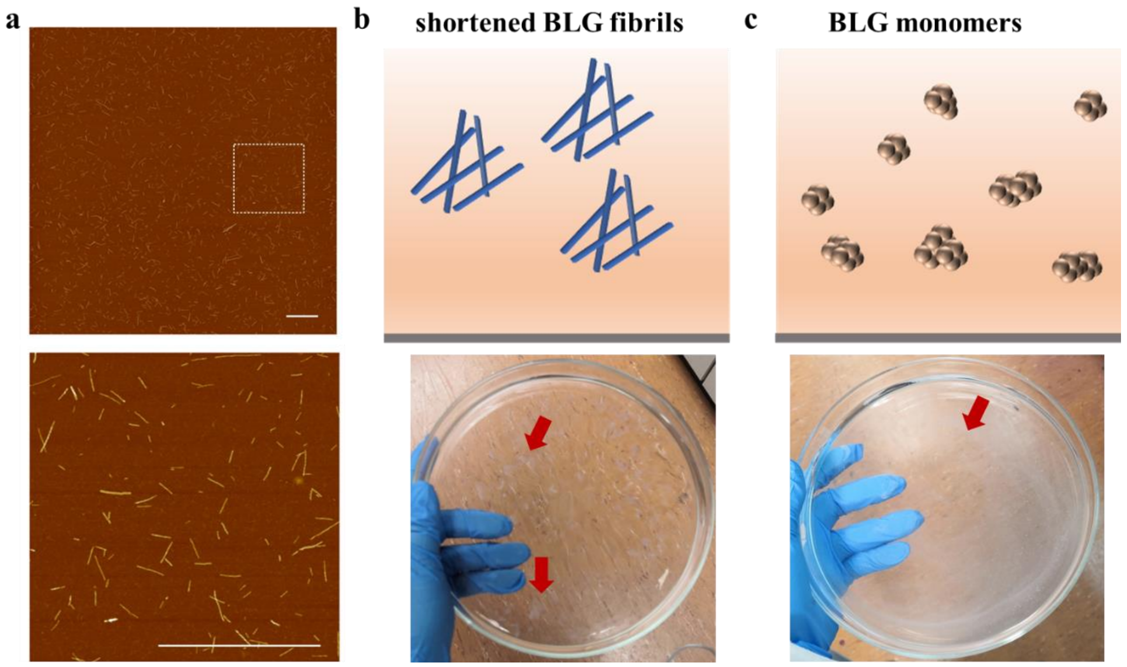

Figure S2. (a) AFM images of shortened BLG fibrils with different magnifications (scale bar:2 μm). (b, c) Schematic illustration (up) and photographs (bottom) that show shortened BLG fibrils (b) and BLG monomers (c) failing to form intact films after spraying phytic acid (only white aggregates can be observed).

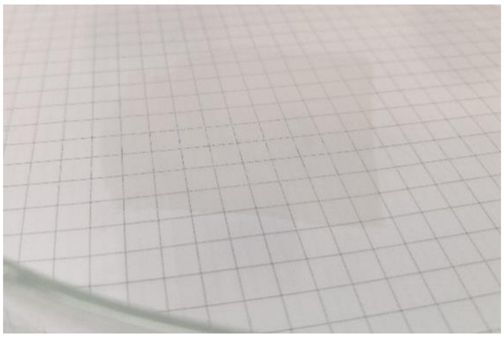

Figure S3. Amyloid film assembled from lysozyme fibril solution (0.5 wt%) using the same protocol. Lysozyme fibril solution was prepared according to the previous work.[2]

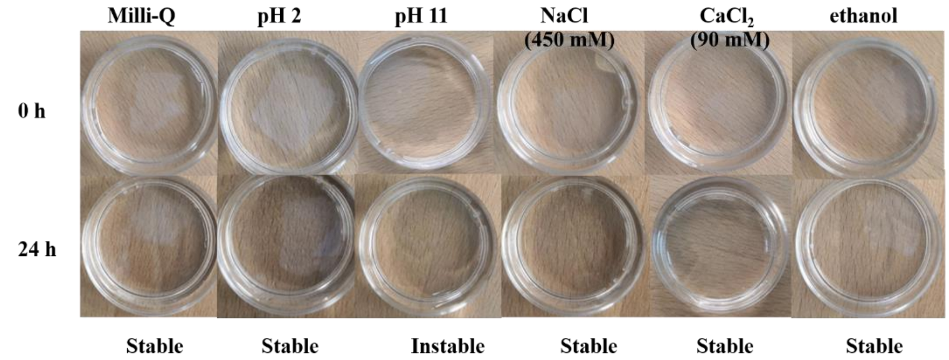

Figure S4. Photographs showing the stability of amyloid film in different solvents.

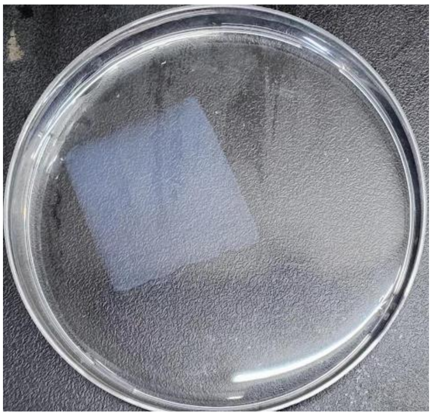

Figure S5. Photograph of the protein film in deionized water after 7 days, that can keep intact.

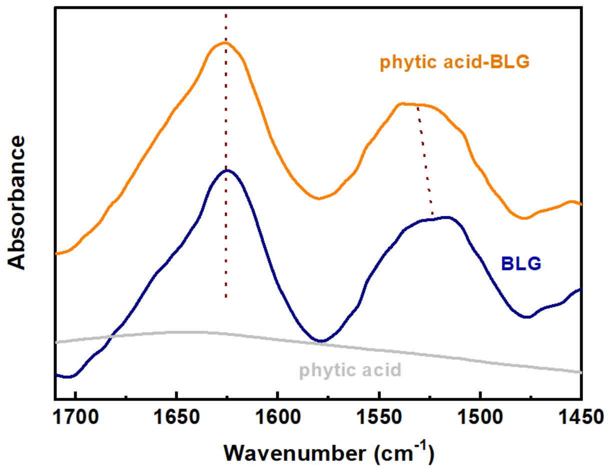

Figure S6. Zoomed-in FTIR spectra of phytic acid, BLG, and phytic acid-BLG.

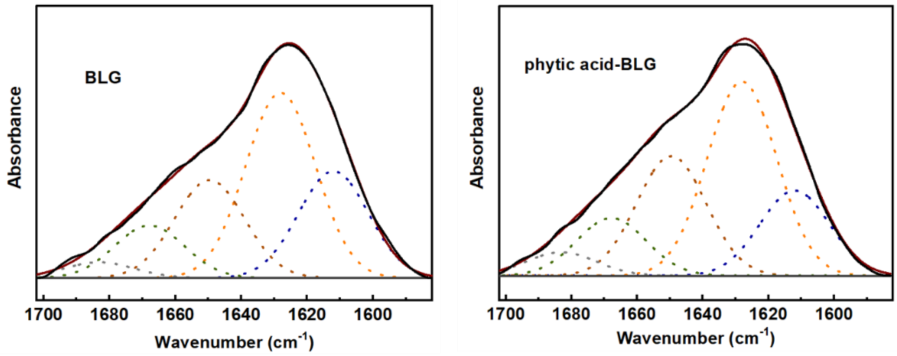

	β-Sheet (1615-1640 cm^{-1})	β-Turn (1660-1700 cm^{-1})	Random coil (1640-1650 cm^{-1})
BLG	63.8	14.8	21.4
phytic acid-BLG	58.2	16.9	24.9

Figure S7. The secondary structure of protein film determined by Fourier deconvolution of amide I band. Summary of the secondary structures of the protein film is presented in the table.

Deconvolution of the amide I group of the spectra (1600-1700 cm^{-1}) using some center peaks (1612, 1628,1650, 1668, and 1683 cm^{-1}) was shown in Figure S7. The two self-deconvolution peaks (1612, and 1628 cm^{-1}) represent β-sheet components and the bands centered at 1652, 1668, and 1683 cm^{-1} are related with random coil and β-turn structure.[3] The amount of β-sheet structure was slightly (<9%) decreased due to the presence of phytic acid.

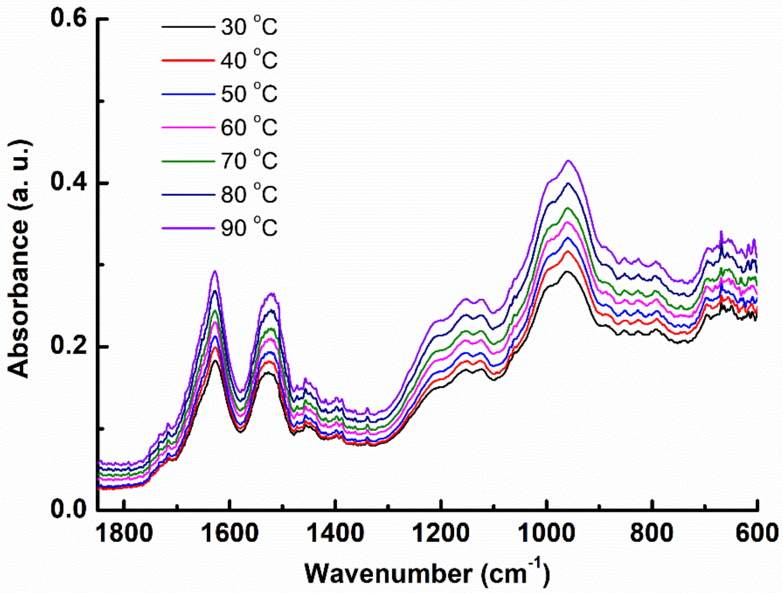

Figure S8. FTIR spectra of phytic acid-BLG at different temperatures.

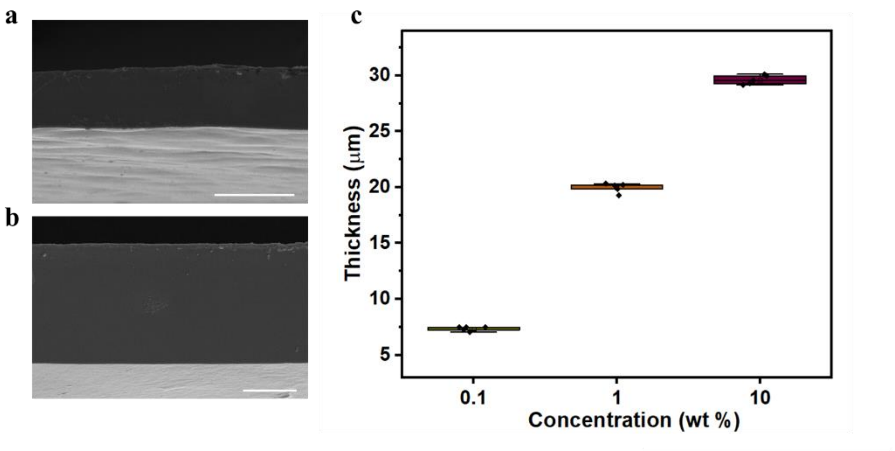

Figure S9. (a, b) SEM images of the cross-section of protein film formed with different phytic acid concentration (a, 0.1 wt%; b, 10 wt%) (scale bar: 10 µm). (c) The thickness of protein films prepared with different phytic acid concentrations (BLG 2 wt%). The thickness of the formed film changes from 7.3 µm to 29.6 µm with different concentrations of phytic acid.

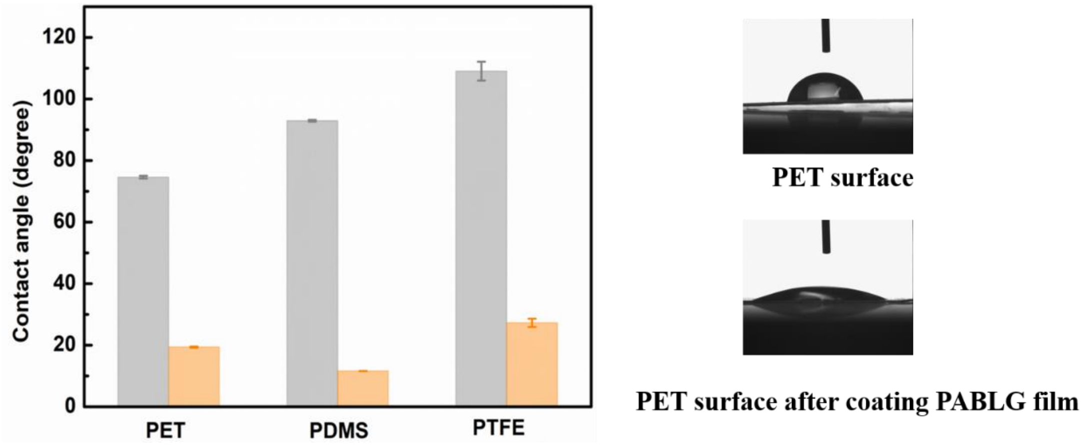

Figure S10. The water contact angles of different substrates before (gray) and after (orange) coating with amyloid film (0.2 wt%).

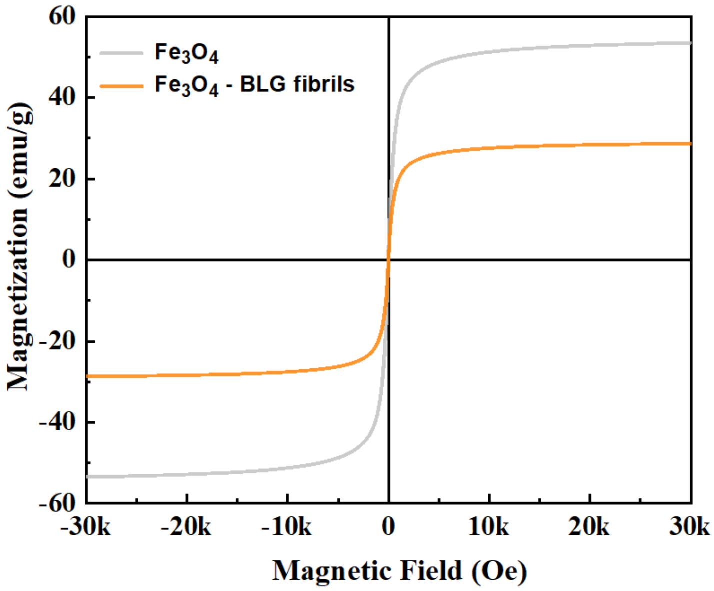

Figure S11. Magnetization of Fe_3O_4 nanoparticles and Fe_3O_4 nanoparticles-coated BLG fibrils.

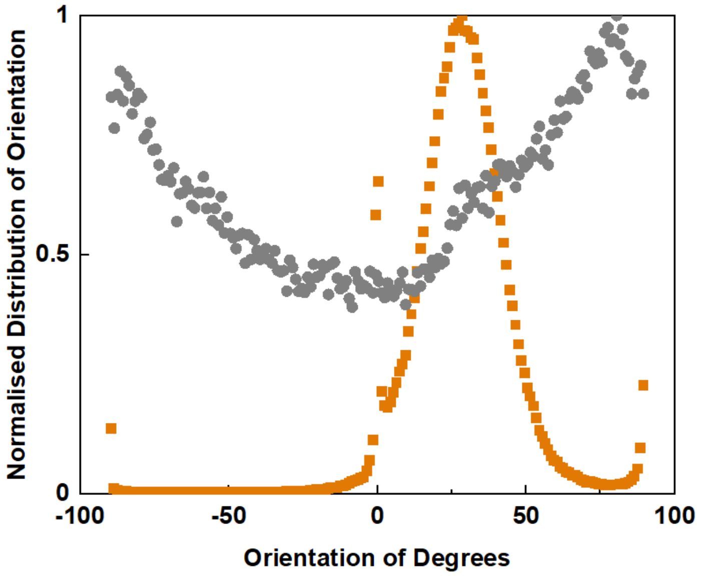

Figure S12. Comparing the directionality of hybrid amyloid films with (orange) and without (gray) exposure to an external magnetic field, with the coherency analysis in the OrientationJ plugin.

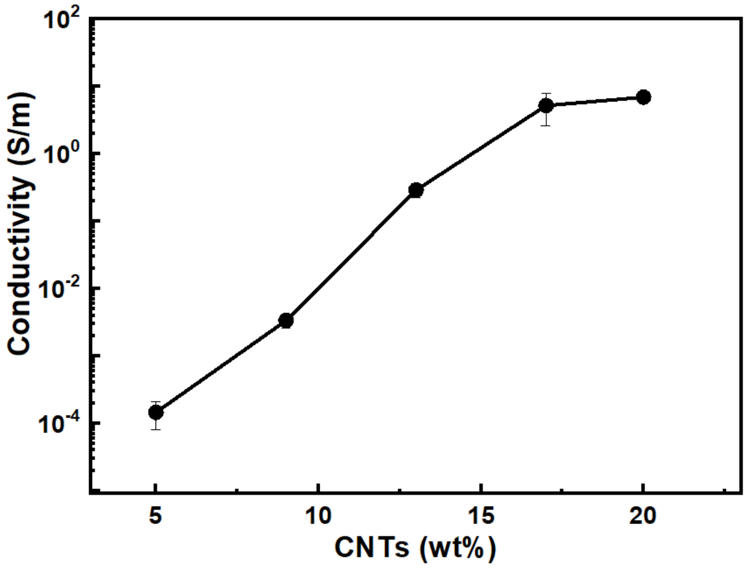

Figure S13. Electrical conductivity of CNTs/BLG films with different CNTs content.

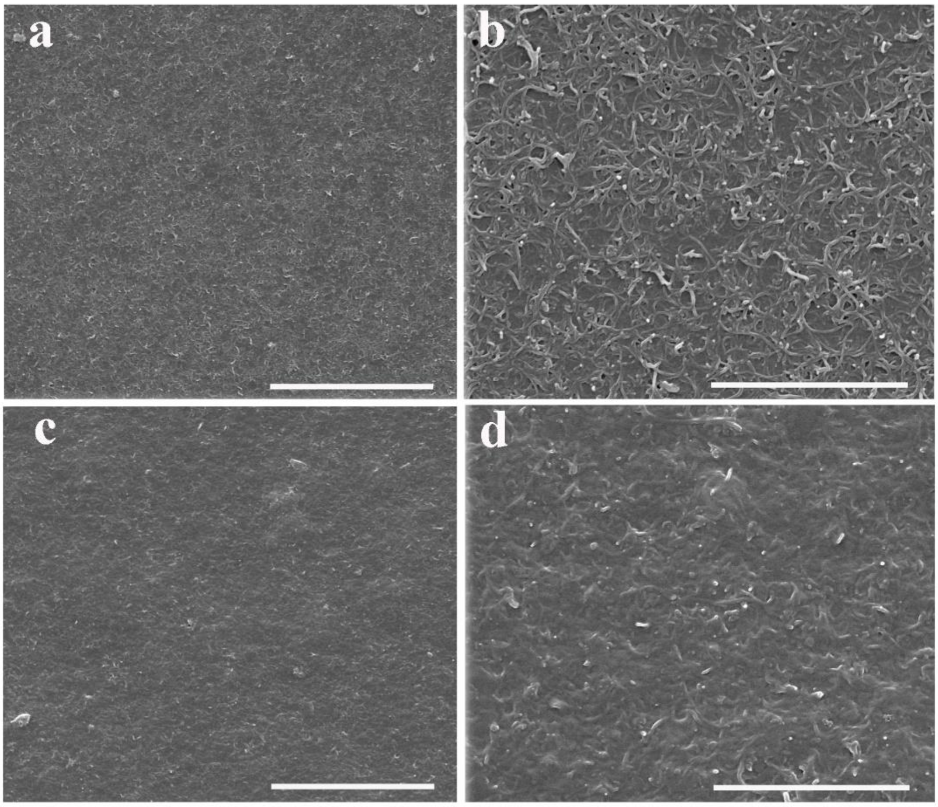

Figure S14. SEM images of CNTs/BLG fibrils (a, b) and phytic acid-CNTs/BLG films (c, d) with different magnifications (Scale bar:10 µm for a and c, and 3 µm for b and d).

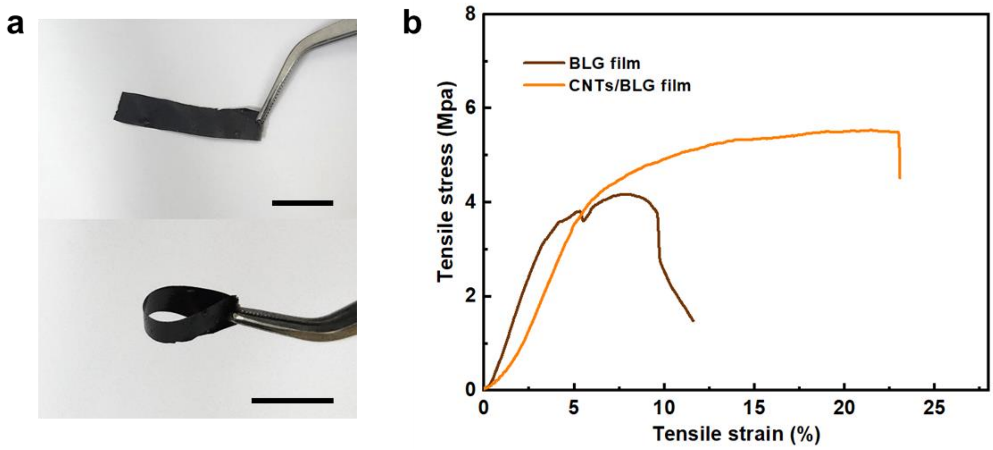

Figure S15. (a) Photograph of CNTs/BLG film (CNTs, 0.62 wt%) (scale bar: 10 mm). (b) Typical strain-stress curves of BLG and CNTs/BLG films.

Tensile tests were performed on a universal testing machine (microcomputer electronic universal material testing machine, QLW-5E) to explore the mechanical properties of the thick protein films (thickness around 30 µm). The BLG film without additives is flexible and ductile material with 4.91 ± 0.65 MPa tensile strength and 8.2 ± 2.2% strain to failure. After introducing the organic CNTs additives, the composite protein film remains flexible. The tensile strength and strain to failure are 5.12 ± 0.89 MPa and 20.7 ± 6.3%. The CNTs as nanofillers has a slightly strengthening and toughening effect on the protein composite film. It should be noted that the thick protein film with the inorganic additives Fe_3O_4 is brittle with cracks, which is not suitable for tensile testing.

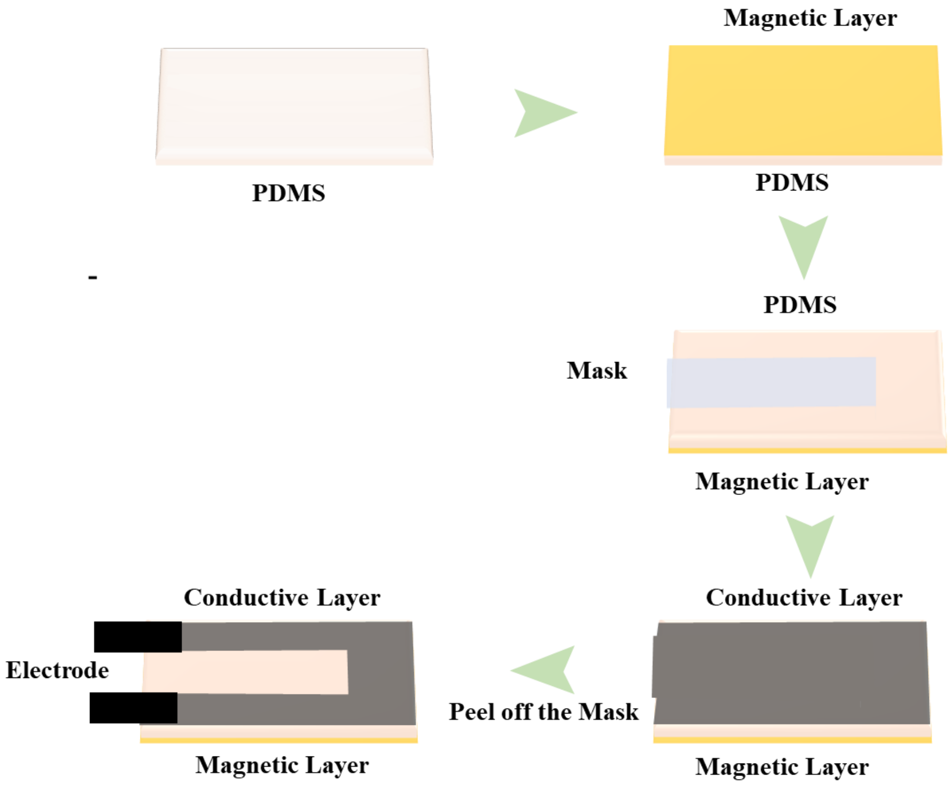

Figure S16. The fabrication process of the magnetic sensor. The magnetic film as the active layer was first transferred onto the top surface of a flexible PDMS substrate. After drying, the middle area of the opposite side of PDMS was covered with a PDMS strip as a mask. Then the conductive layer was transferred onto the surface of PDMS film. After peeling off the mask, two sides of conductive layers were connected with flexible copper foil as electrode, by using a conductive silver paste to eliminate the contact resistance and obtain a steady signal output.

Reference

[1] J. J. V. Sreenath Bolisetty, Jozef Adamcik, and Raffaele Mezzenga, ACS Nano 2013, 7, 6146-6155.
[2] Y. Cao, S. Bolisetty, G. Wolfisberg, J. Adamcik, R. Mezzenga, Proc. Natl. Acad. Sci. U. S. A. 2019, 116, 4012-4017.
[3] W. Lee, I. Kim, S. W. Lee, H. Lee, G. Lee, S. Kim, S. W. Lee, D. S. Yoon, Macromolecular Research 2016, 24, 868-873.
